# Circulating Plasma miRNA-548L as Novel Predictive Biomarker of Radiotherapy-Induced Severe Oral Mucositis in Patients with Laryngeal Cancer

**DOI:** 10.3390/genes17050578

**Published:** 2026-05-18

**Authors:** Marcin Mazurek, Anna Brzozowska, Teresa Małecka-Massalska, Tomasz Powrózek

**Affiliations:** 1 Department of Human Physiology of the Chair of Preclinical Sciences, Medical University of Lublin, 20-080 Lublin, Poland; 2 Department of Radiotherapy, St. John of Dukla Lublin Region Cancer Center, 20-090 Lublin, Poland

**Keywords:** laryngeal cancer, oral mucositis, miRNA-548L, radiotherapy

## Abstract

**Background/Objectives:** Oral mucositis (OM) is a common complication in laryngeal cancer (LC), during radiotherapy (RT), significantly affecting patient outcomes. Identifying sensitive biomarkers to predict OM severity is therefore crucial. MicroRNAs, which regulate inflammatory pathways involved in OM, are promising candidates. This retrospective study aimed to evaluate plasma miRNA-548L as a predictive biomarker for the occurrence and severity of OM in LC patients undergoing RT. **Methods:** The expression levels of the selected miRNAs were analyzed in plasma samples obtained from 76 LC patients prior to the initiation of RT. Bioinformatics analyses were performed to identify molecular pathways regulated by miRNA-548L and their potential link to the pathogenesis of OM. **Results:** Significantly decreased levels of the studied miRNA were observed in the plasma of LC patients who developed severe OM after the IV^th^ (*p* < 0.001), V^th^ (*p* = 0.039), VI^th^ (*p* < 0.001), and VII^th^ (*p* < 0.001) cycles of RT. Additionally, the expression of miRNA-548L enabled reliable differentiation between patients who developed severe OM during the IV^th^ (AUC = 0.81, *p* < 0.001), V^th^ (AUC = 0.77, *p* < 0.001), VI^th^ (AUC = 0.82, *p* < 0.001), and VII^th^ (AUC = 0.86, *p* < 0.001) weeks of treatment. Importantly, lower expression of miRNA-548L (HR = 3.12; *p* = 0.010) was significantly associated with shorter median overall survival (OS). **Conclusions:** Pretreatment plasma miRNA-548L shows potential as a biomarker for predicting severe OM in LC patients undergoing RT. Notably, reduced miRNA-548L expression is associated with shorter OS and may help stratify patients by OM severity.

## 1. Introduction

Among head and neck cancers (HNCs), laryngeal carcinoma (LC) represents the most prevalent malignancy, accounting for approximately 30–40% of all HNC cases. LC primarily arises from the epithelial lining of the laryngeal mucosa and is strongly associated with prolonged exposure to carcinogenic agents [1,2]. Major etiological factors include tobacco smoking, excessive alcohol consumption, and infection with high-risk human papillomavirus (HPV) strains [3]. More than 90% of LCs originate from the mucosal epithelium, with well-differentiated squamous cell carcinoma constituting the predominant histopathological subtype [4].

Radiotherapy (RT) is used in approximately 75% of HNC cases and is frequently administered in combination with other therapeutic modalities, including surgery and chemotherapy (CTH). Despite its clinical efficacy, RT particularly when combined with CTH can induce damage to both malignant and normal tissues, with the highly radiosensitive mucosal lining of the oral cavity and pharynx being especially vulnerable. This tissue injury commonly results in oral mucositis (OM), a prevalent and debilitating complication of irradiation [5]. OM typically manifests within 2–4 weeks following the initiation of RT and may persist for up to five weeks following treatment completion [6]. The incidence of OM varies according to treatment modality, affecting approximately 30–40% of patients receiving CTH, 60% of those undergoing RT alone, and up to 90% of patients treated with concurrent chemoradiotherapy (CRT) [7,8,9]. The development of OM is associated with increased hospitalization rates, poor quality of life, and in approximately two-thirds of patients, necessitates dose reduction or interruption of RT. These complications may ultimately compromise therapeutic efficacy and adversely affect patient survival [10]. The pathophysiology of OM involves an inflammatory cascade initiated by ionizing radiation-induced mucosal injury [9]. Ultimately, this process leads to ulceration, erythema, edema, epithelial atrophy and pseudomembrane formation. During the ulcerative phase, the transcription nuclear factor kappa beta (NFκ-B) pathway becomes activated, resulting in the release of multiple proinflammatory mediators such as interleukin-1β (IL-1β), interleukin-6 (IL-6), and tumor necrosis factor-alpha (TNF-α). These cytokines further activate macrophages and upregulate genes involved in inflammatory responses, including cyclooxygenase-2 (COX-2), mitogen-activated protein kinase (MAPK), and tyrosine kinase signaling pathways. The resulting signaling cascade promotes tissue degradation through activation of matrix metalloproteinases (MMP-1 and MMP-3) within the lamina propria and epithelial layers [11,12,13].

The substantial clinical burden of OM underscores the need for reliable biomarkers capable of predicting its onset and severity, thereby enabling early intervention and individualized risk stratification. Although direct genetic manipulation remains technically challenging, modulation of protein-coding genes and regulatory pathways may offer a strategy to attenuate OM progression, even among patients with similar demographic and clinical characteristics. Among the most promising biomarker candidates are microRNAs (miRNAs), non-coding RNA molecules that regulate gene expression at the post-transcriptional level. miRNAs are involved in a wide range of biological processes, including inflammation, metabolism, and therapeutic response [14,15]. Circulating plasma miRNAs have emerged as potential indicators of radiation-induced toxicities, including OM, in patients with HNC. Nevertheless, robust clinical evidence remains limited, and comprehensive studies evaluating their predictive value for radiation-induced OM are lacking. Several miRNAs, including miRNA-141, miRNA-200a, miRNA-200b, and miRNA-200c, have been associated with the pathogenesis of RT-induced OM in preclinical models, with miRNA-200c expression notably upregulated in irradiated normal human keratinocytes (NHKs) [16]. Furthermore, elevated plasma levels of miRNA-425-5p, -21-5p, -106b-5p, and -93-5p have been observed in irradiated HNC patients [17]. However, the expression patterns and functional role of miRNA-548L in HNC remain largely unexplored.

The present study aimed to investigate the association between miRNA-548L expression levels and the incidence and severity of OM in patients receiving radiotherapy-based treatment for advanced LC. A secondary objective was to assess the potential prognostic value of miRNA-548L in this patient population.

## 2. Material and Methods

### 2.1. Study Group

This retrospective study included 76 patients (64 men and 12 women, mean age: 63 years) with advanced or locally advanced LC. All participants were recruited and received RT-based treatment at the Department of Oncology, Medical University of Lublin, between 2014 and 2017. The study protocol was approved by the Bioethical Commission of the Medical University of Lublin (approval number: KE-0254/64/2017) and was conducted in accordance with the ethical principles outlined in the 1964 Declaration of Helsinki and its subsequent amendments. Written informed consent was obtained from all participants prior to inclusion in the study. Disease staging was performed according to the 8th edition of the TNM classification. Inclusion criteria were: age ≥ 18 years, both genders, confirmed diagnosis of advanced LC, and completion of a full course of RT or CRT. Exclusion criteria included a prior history of malignancy, coexisting cancers, autoimmune disorders, and active infections.

### 2.2. Treatment Protocol

All patients were treated using an ONCOR (Siemens, München, Germany) linear accelerator with an intensity-modulated radiotherapy (IMRT) approach. Radiation doses ranged from 54 to 70 Gy, delivered in 2 Gy daily fraction, five days per week. Patients with advanced disease, including enlarged lymph nodes and primary tumors, received a total of 70 Gy administered in 35 fractions. Postoperative patients with high-risk volumes received 66 Gy in 33 fractions. Radiation doses for low-risk and intermediate-risk groups were 54 Gy and 60 Gy, respectively, with elective lymph nodes receiving either 54 Gy or 60 Gy as indicated. In addition to IMRT, selected patients received 1–4 cycles of CTH consisting of cisplatin (100 mg/m^2^ on day 1) and 5-fluorouracil (1000 mg/m^2^ per day, continuous infusion on days 1–5). The concurrent chemoradiation course involved the administration of cisplatin in the dose of 100 mg/m^2^ every 21 days. The complete course of therapy for all patients was seven weeks. All patients completed the course of RT with the prescribed dose of IMRT without interruption.

### 2.3. The Assessment of OM

OM was evaluated at baseline and subsequently on a weekly basis throughout RT, with assessments conducted in the presence of both a laryngologist and a radiotherapist at each time point. OM severity was graded according to the Radiation Therapy Oncology Group/European Organization for Research and Treatment of Cancer (RTOG/EORTC) criteria and classified as follows: 0—none, 1—mild, 2—moderate, 3—severe, and 4—life-threatening. The assessors were blinded to miRNA-548L expression levels and all laboratory results.

### 2.4. miRNA Expression Analysis

Prior to the initiation of RT, 5 mL of whole blood was collected from each participant. Samples were centrifuged at 1000× *g* for 15 min, and platelet-poor plasma was collected within 30 min of centrifugation. Plasma samples were stored at −80 °C until genetic analysis. miRNA was then extracted from 200 μL of plasma using a column-based method according to the manufacturer’s instructions (miRNeasy Serum/Plasma Kit, Qiagen GmbH, Hilden, Germany). The isolated miRNA was reverse-transcribed into complementary DNA (cDNA) using the TaqMan Advanced miRNA cDNA Synthesis Kit (Thermo Fisher Scientific, Waltham, MA, USA). Relative miRNA expression levels were measured at the same time point, between 24 and 72 h before RT. Quantitative PCR amplification was performed on a StepOnePlus system (Applied Biosystems, Foster City, CA, USA) using TaqMan Fast Advanced Master Mix (Thermo Fisher Scientific, Waltham, MA, USA) and the TaqMan Advanced miRNA Assay probe specific for miRNA-548L (Assay name: hsa-miRNA-548L; Assay ID: 479391_mir; Thermo Fisher Scientific, Waltham, MA, USA). All samples were analyzed in triplicate. The expression levels of the studied miRNAs were normalized to miRNA-26a-3p as an internal control (Assay name: hsa-miRNA-26a-3p; Assay ID: 478787mir; ThermoFisher Scientific, Waltham, MA, USA) using 2^−ΔΔCt^ and 2^−ΔCt^ formulae.

### 2.5. Statistical Analysis

Statistical analyses were performed using MedCalc v.15.8 software (MedCalc Software, Ostende, Belgium). Differences in miRNA expression according to clinical and demographic factors, as well as OM severity, were evaluated using the Mann–Whitney U test. Odds ratios (ORs) with 95% confidence intervals (CIs) were calculated to assess the likelihood of radiation-induced OM based on clinical, demographic, and miRNA expression variables. Multivariable analysis of OM risk, accounting for demographic, clinical, and genetic factors, was conducted using logistic regression. The backward elimination method was employed to select statistically significant results from the univariable analysis for inclusion. Receiver operating characteristic (ROC) curves were generated using pretreatment values toto determine optimal cut-off values and assess the diagnostic performance of the investigated miRNA for identifying grade 3 OM in irradiated LC patients. Overall survival (OS) was analyzed using the Kaplan–Meier method with log-rank tests, and variables influencing OS were further evaluated via Cox proportional hazards regression.

Gene targets of miRNA-548L were predicted using three databases: TargetScan Human 8.0 (criterion: weighted context score < −0.3), miRDB (criterion: prediction score > 85), and miRmap (criterion: miRmap score > 85). Functional enrichment analysis was performed using the SRplot (Science R plot) software, the PANTHER classification system, and the Reactome pathway browser to identify enriched Gene Ontology (GO) terms, Kyoto Encyclopedia of Genes and Genomes (KEGG) pathways, and biological processes associated with miRNA-548L target genes. Subsequently, selected enrichment terms were evaluated for their potential relevance to the etiology of OM. Enrichment terms with an adjusted *p*-value (*p*. adjusted) < 0.05 were considered statistically significant.

## 3. Results

### 3.1. Characteristics of Patients

Males constituted the majority of the study cohort (84.2%). Histopathological evaluation revealed that 25% of patients were diagnosed with stage III disease, whereas 75% presented with stage IV LC, according to the 8th edition of the TNM classification. A history of alcohol consumption was reported in 44.7% of patients. Tobacco use was highly prevalent, with 72.4% of the cohort identified as smokers; among them, 89.1% were current smokers at the time of diagnosis. Comprehensive clinical and demographic data for all participants are summarized in Table 1.

We further examined the association between miRNA-548L expression levels and selected clinical and demographic variables in patients with LC. Stage IV of the disease was significantly associated with lower miRNA-548L expression compared to stage III (median: 2.96 vs. 5.90; *p* < 0.001). Additionally, patients with poorer performance status, defined as >1 according to the ECOG-WHO scale, exhibited significantly reduced miRNA-548L expression relative to those with better performance status (median: 4.93 vs. 8.91; *p* = 0.024) (Table S1).

### 3.2. Relationships Between Expression of the Studied miRNA and Severity of OM

Patients who developed moderate OM (grade 2) after completion of the III^rd^ cycle of RT exhibited significantly lower expression of the investigated miRNA compared with those presenting with mild OM (grade 1) (median: 4.21 vs. 5.58; *p* = 0.038). Beginning with the assessment conducted after the IV^th^ week of irradiation and continuing through weeks Vth, VI^th^ and VII^th^, patients with grade 3 OM consistently demonstrated reduced miRNA-548L expression relative to those with grades 1–2 OM (Table 2).

### 3.3. Pretreatment Expression of miRNA as Predictors of Severe Grade of OM

**Figure 1:**
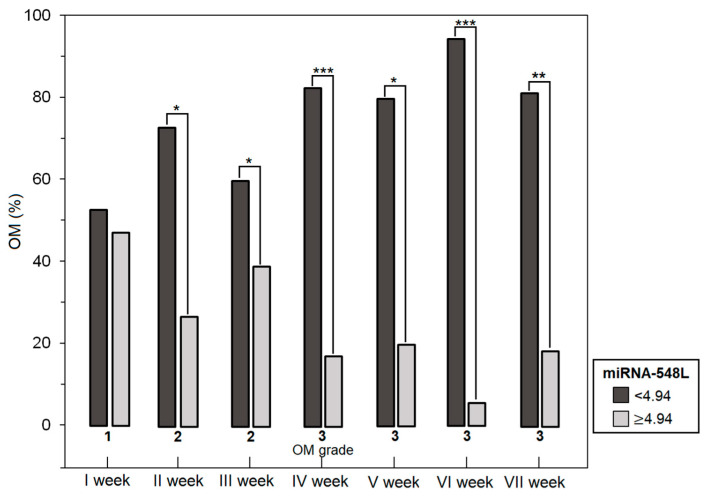
Changes in the incidence of higher grade OM during radiotherapy in LC patients according to miRNA-548L expression levels (***—*p* < 0.001; **—*p* < 0.01; *—*p* < 0.05).

Based on the pretreatment median expression level of miRNA-548L (median: 4.94), patients with LC were stratified into two groups: low and high miRNA-548L expression. Low expression was observed in 39 patients (51.3%), while 37 patients (48.7%) were classified as having high expression levels. From the IV^th^ week of RT onward (weeks IV–VII), a significantly higher proportion of patients with low miRNA-548L expression developed grade 3 OM compared with those exhibiting high expression levels. The differences in the proportion of severe OM cases during subsequent weeks of RT according to miRNA-548L expression status are presented in Figure 1.

**Figure 2:**
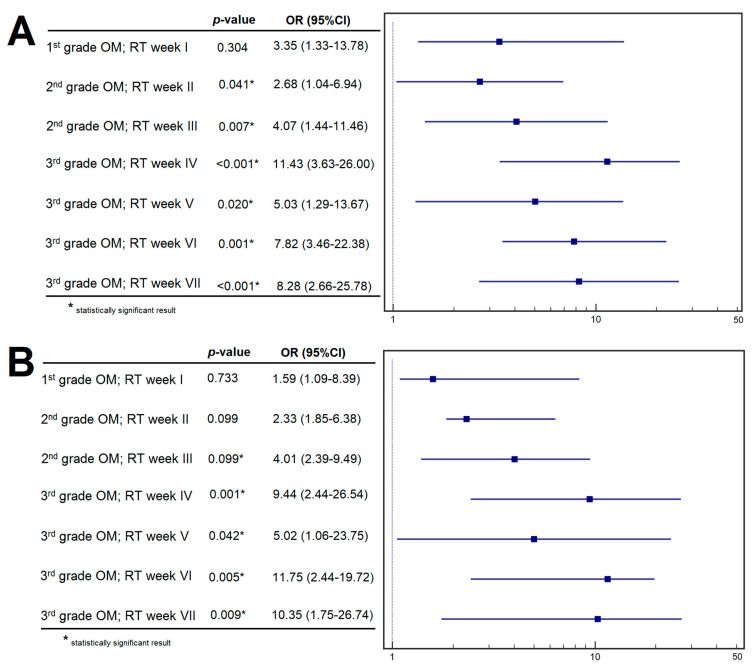
Plasma miRNA-548L as a predictor of OM in LC patients during subsequent weeks of RT: (**A**) univariate analysis; (**B**) multivariate analysis.

Patients with decreased miRNA-548L expression demonstrated a significantly higher probability of developing moderate OM following the II^nd^ (OR = 2.68; *p* = 0.041) and III^rd^ (OR = 4.07; *p* = 0.007) cycles of RT. Beginning in the IV^th^ week of RT, low miRNA-548L expression level was associated with a markedly increased risk of grade 3 OM, with ORs of 11.43 (*p* < 0.001), 5.03 (*p* < 0.001), 7.82 (*p* = 0.001), and 8.82 (*p* < 0.001) for weeks IV, V, VI, and VII, respectively (Figure 2A).

Multivariable logistic regression analysis demonstrated a significantly increased risk of grade 3 OM after the III^rd^ week of RT in patients with low miRNA-548L expression (OR = 4.01; *p* = 0.009). Moreover, from the completion of the IV^th^ week through the end of RT, decreased miRNA levels remained independently associated with a higher probability of severe OM, with ORs of 9.44 (*p* = 0.001), 5.02 (*p* = 0.042), 11.57 (*p* = 0.005), and 10.35 (*p* = 0.009) for weeks IV, V, VI, and VII, respectively (Figure 2B). Detailed results of the univariable and multivariable analyses are provided in Tables S2 and S3.

### 3.4. Diagnostic Value of Pretreatment miRNA-548L in Distinguishing Between LC Patients with Different Grades of OM

The predictive performance of miRNA-548L, measured before the initiation of RT, varied across different time points of treatment in relation to the development of severe OM. During the III^rd^ week of treatment, miRNA-548L demonstrated moderate diagnostic performance for predicting severe OM, with a sensitivity of 80% and a specificity of 55.7% (AUC = 0.67; *p* = 0.019). Following completion of the IV^th^ week of RT, miRNA exhibited improved diagnostic accuracy in discriminating patients with severe OM, achieving a sensitivity of 79.3% and a specificity of 74.5% (AUC = 0.81; cut-off < 5.41; *p* < 0.001). Furthermore, in week V, miRNA-548L demonstrated significant discriminatory power in distinguishing between severe and mild OM, achieving a sensitivity of 94.4% and a specificity of 62.1% (AUC = 0.77; *p* < 0.001). In week VI, miRNA also showed significant diagnostic value for identifying severe OM among LC patients, with a sensitivity of 72.2% and a specificity of 100% (AUC = 0.82; *p* < 0.001). The best diagnostic accuracy was observed after the VII^th^ cycle of RT (AUC = 0.86; *p* < 0.001). Detailed results are presented in Table 3.

### 3.5. Relationship Between the Expression Plasma Levels of the miRNA-548L and Overall Survival

Univariate survival analysis using the Kaplan–Meier method with log-rank testing demonstrated that patients with T4 tumors and more advanced disease (stage IVA–C) had a significantly higher risk of early mortality, with HRs of 3.10 (*p* < 0.001) and 3.75 (*p* < 0.001), respectively, compared with other patients. Additionally, patients with decreased plasma miRNA-548L expression exhibited significantly shorter median OS than those with higher miRNA levels (median OS: 18 months vs. 36 months; HR = 3.12; *p* = 0.010).

**Figure 3:**
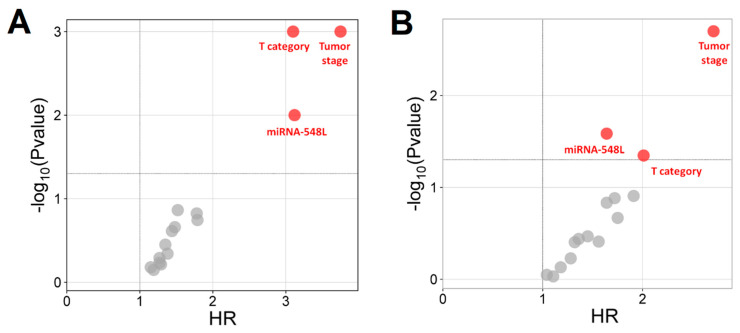
Independent prognostic factors affecting LC patients’ survival: (**A**) univariate analysis; (**B**) multivariate analysis (red dots indicate significant factors and grey dots insignificant factors).

Multivariate survival analysis using the Cox proportional hazards model identified T4 tumor category (HR = 2.01; *p* = 0.045), advanced TNM stage (IVA–C) (HR = 2.71; *p* = 0.002), and decreased plasma miRNA-548L expression level (HR = 1.64; *p* = 0.026) as independent factors significantly affecting OS. During the follow-up period, 42 of 76 patients (55.3%) died. Mortality was significantly higher among patients with low plasma miRNA-548L levels (27/39; 69.2%) compared with those with higher expression (15/37; 40.5%; *p* = 0.039). Comprehensive results from both univariate and multivariate OS analyses are presented in Table S4, and the associations between selected demographic, clinical, and molecular variables and OS are illustrated in Figure 3.

### 3.6. Identification of miRNA-548L Targets and Their Association with OM Pathogenesis

**Figure 4:**
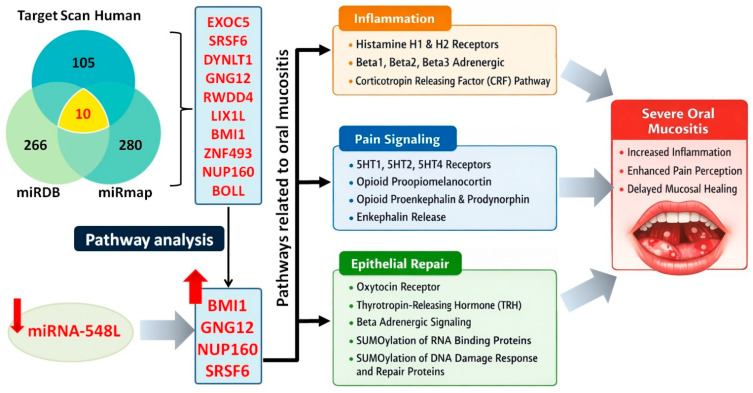
The process of identifying gene targets for miRNA-548L and the selected genes involved in processes related to the etiology of OM. Signaling pathways regulated by genes targeted by miRNA-548L were classified into three categories: inflammation, pain signaling, and endothelial repair (based on Reactome pathway browser, PANTHER classification system, and Gene Ontology analyses).

Applying the specified search criteria, 105 (TargetScan Human 8.0), 266 (miRDB), and 280 (miRmap) gene targets were computationally predicted for miRNA-548L. Ten genes were common to all three databases: *EXOC5*, *SRSF6*, *DYNLT1*, *GNG12*, *RWDD4*, *LIX1L*, *BMI1*, *ZNF493*, *NUP160*, and *BOLL*. To explore the potential functional roles of these candidates, a pathway enrichment analysis related to OM was subsequently performed for this gene set. Four genes, *BMI1*, *GNG12*, *NUP160*, and *SRSF6*, showed significant enrichment involvement in biological pathways potentially associated with OM etiology. Identified enrichment terms were categorized into three principal OM-related groups: inflammation, pain signaling, and epithelial repair. Within the inflammation category, the histamine receptor signaling pathway, β-adrenergic receptor signaling pathway, and corticotropin-releasing hormone signaling pathway were identified as significantly associated with OM and might be mediated by *GNG12*. Similarly, *GNG12* was predicted to be potentially involved in several pain-related pathways that may contribute to OM development, including the 5-HT receptor signaling pathway, opioid receptor signaling pathway, and enkephalin release. Regarding epithelial repair, biological processes potentially involving *GNG12*, *BMI1*, and *NUP160* included the oxytocin receptor signaling pathway, thyrotropin-releasing hormone signaling pathway, β-adrenergic receptor signaling pathway, and SUMOylation of DNA damage response and repair proteins (Figure 4). Additionally, based on GO analysis, *BMI1* and *SRSF6* were predicted to be associated with processes such as wound healing, regulation of epithelial cell apoptosis, and regulation of keratinocyte proliferation. No significant enrichment terms were identified in the KEGG analysis. A detailed summary of the PANTHER classification system, Reactome pathway browser, and GO analyses is presented in Tables S5 and S6. It should be noted, however, that these associations remain predictive and require further experimental validation.

## 4. Discussion

Approximately 85% of HNC patients receive RT as part of their treatment [18]. RT is also the primary therapy for LC, but it carries a substantial risk of both acute and late toxicities, which affect nearly all patients following curative treatment [19]. OM, an immediate adverse effect of RT, occurs in the majority of HNC patients, including those with LC, with reported incidence ranging from 59.4% to 100% [20,21]. Tumor-promoting inflammation and treatment-related toxicity are considered key contributors to OM development. Current methods for monitoring and predicting OM are often inadequate. Given their stability and role in disease progression, miRNAs are increasingly being explored as diagnostic and prognostic biomarkers in LC [22].

Circulating blood-derived miRNAs are increasingly recognized as diagnostic and prognostic biomarkers in cancer due to their stability and regulatory roles in disease progression. They modulate genes and pathways affecting tumor radiosensitivity and responses to RT, including oxidative stress, DNA repair, and apoptosis, enabling prediction of treatment outcomes [23,24,25]. Experimental studies have demonstrated selective changes in miRNA expression following irradiation. For example, Wagner-Ecker et al. reported that a dose of 2 Gy ionizing radiation induced upregulation of let-7g, miRNA-20a, and miRNA-21 and downregulation of miRNA-125a, -127, and -189 in human dermal microvascular endothelial cells, affecting radiosensitivity and cell viability [26]. Tao et al. observed significant increases in miRNA-141 and -200a/b/c expression in murine tongue tissue and normal human keratinocytes after irradiation, accompanied by elevated proinflammatory cytokines, implicating these miRNAs in radiation-induced tissue injury [16]. Despite these advances, no studies have investigated the role of miRNA-548L in RT-related OM. Previous work has highlighted other miRNAs, such as miRNA-18b-3p, as potential biomarkers of RT/CRT-induced OM, with elevated circulating levels correlating with the extent of irradiated oral mucosa [27]. In contrast, our study demonstrated a significant decrease in plasma miRNA-548L levels from the fourth to the seventh week of RT in patients who developed severe OM. Low miRNA-548L expression was strongly associated with an increased probability of grade 3 OM, beginning in the fourth week of treatment. ROC analysis confirmed the predictive value of miRNA-548L for early detection of severe OM, showing high sensitivity (approaching 100% by the fifth week) and increasing specificity in subsequent weeks, reaching 100% in the sixth week. Our results suggest that miR-548L may enable the reliable identification of patients at high risk and facilitate timely therapeutic intervention. Overall, miR-548L emerges as a promising circulating plasma biomarker for monitoring and predicting RT-induced OM in patients with LC.

Conducted bioinformatics analyses indicated that decreased miRNA-548L expression can be associated with upregulation of *BMI1*, *GNG12*, *NUP160*, and *SRSF6*. These candidate targets were prioritized because they converge on the NF-κB signaling pathway, a central regulator of the inflammatory cascade in RT-induced injury. For instance, in glioma cell lines (LN229 and A172), elevated *BMI1* expression activates the NF-κB pathway and increases MMP-9 mRNA levels [28], while in patients with inflammatory bowel disease, high *BMI1* correlates with elevated plasma IL-6 and TNF-α [29]. Similarly, overexpression of GNG12 and NUP160 has been shown to enhance NF-κB activation and promote the production of IL-1β, IL-6, and TNF-α in various cellular models [30,31]. Furthermore, overexpression of *SRSF6* alters splicing and upregulates inflammation-related genes such as *TNFA*, *IL1F*, and *Cxcl2*, leading to higher proinflammatory cytokine production [32]. Collectively, while the involvement of these genes in inflammation has been documented in other pathological contexts, our data allow us to hypothesize that miRNA-548L downregulation might contribute to OM-related inflammatory processes through the potential upregulation of these predicted targets. However, this mechanistic link remains hypothetical and requires direct experimental validation to confirm whether these interactions occur in oral mucosal tissues during RT. In addition to inflammation, bioinformatics analyses revealed that miRNA-548L may also be involved in pathways regulating pain signaling and endothelial repair, both of which are significantly associated with the pathophysiology of OM. In the present study, the identified miRNA-548L target genes are based solely on in silico predictions, as no experimental validation has been reported to date for BMI1, GNG12, NUP160, or SRSF6. Therefore, these findings should be considered hypothesis-generating and require further experimental confirmation to establish their definitive roles in OM.

Several miRNAs have been reported as prognostic biomarkers in LC so far. Elevated miRNA-146a-5p expression was associated with significantly shorter OS (*p* = 0.018), while reduced miRNA-449a-5p correlated with poorer OS (*p* = 0.029). Multivariate analyses identified increased expression of miRNA-34-5p, -378c-5p, -146a-5p, and -34a-5p as independent predictors of shorter OS (HR = 3.47, *p* = 0.047; HR = 3.77, *p* = 0.035; HR = 9.67, *p* = 0.003; HR = 6.76, *p* = 0.006, respectively) [33]. Elevated miRNA-23a, -93-5p, and -210-3p were also linked to poorer 5-year survival outcomes [34,35]. Conversely, higher miRNA-195 expression was associated with longer OS (median: 57 vs. 39 months; *p* = 0.015) [36]. In the present study, low plasma miRNA-548L expression was associated with a more than three-fold increased risk of shorter OS (median: 18 vs. 36 months; HR = 3.12; *p* = 0.010), highlighting its potential as a prognostic biomarker in LC patients.

This study represents one of the first comprehensive evaluations of circulating miRNA-548L in patients with LC undergoing RT, highlighting its potential as both a diagnostic and predictive biomarker for OM. Our findings expand the understanding of miRNA-based patient stratification and provide a rationale for future large-scale studies incorporating additional molecular determinants. Integration of miRNA-548L into OM risk prediction models may improve personalized oncology approaches. Circulating miRNAs can be reliably measured via minimally invasive liquid biopsy during routine laboratory procedures, offering a rapid, cost- and time-efficient method. Early detection of low miRNA-548L levels may identify patients at higher risk of severe RT-induced OM, enabling timely preventive interventions and optimized supportive care.

This study has several limitations. It was conducted at a single center, and its retrospective design and relatively small sample size may limit the generalizability of the findings. The absence of external validation further constrains the applicability of the results. Moreover, the study may not have fully accounted for potential confounding variables, and selection bias could have influenced the observed results. Another limitation of the present study is the lack of analysis evaluating the association between OM severity and OS. Nevertheless, the cohort was relatively homogeneous in terms of tumor stage and treatment modality, which reduces clinical heterogeneity and strengthens internal consistency. Importantly, our analysis demonstrates the potential clinical utility of miRNA-548L in predicting severe OM in LC patients undergoing RT. Importantly, bioinformatics findings are based on in silico predictions and should be interpreted with caution, as experimental validation is required to confirm the proposed miRNA–target interactions and their functional relevance. These findings may have important clinical implications for early risk stratification, facilitating individualized treatment planning and intensified monitoring of high-risk patients. Future multicenter, prospective studies with larger cohorts are needed to validate these results.

## 5. Conclusions

Circulating miRNA-548L may enable monitoring and prediction of the risk of severe OM in LC patients undergoing RT. Pretreatment plasma levels of this miRNA may help distinguish patients likely to develop severe OM from those with milder forms. Importantly, low miRNA-548L expression was also associated with significantly shorter OS in LC patients. Moreover, bioinformatics analyses indicated that miR-548L is closely linked to the pathophysiology of OM.

## Figures and Tables

**Table 1:** Baseline characteristics of the study group.

Factor		*n* = 76 (100%)
Gender	Male	64 (84.2%)
	Female	12 (15.8%)
Age	Mean (range)	63 (42–87)
	≥63 years	39 (51.3%)
	<63 years	37 (48.7%)
T category	T1	2 (2.6%)
	T2	11 (14.5%)
	T3	23 (30.3%)
	T4	40 (52.6%)
N category	N0	24 (31.6%)
	N1	9 (11.8%)
	N2	38 (50%)
	N3	5 (6.6%)
M category	Mx	3 (3.9%)
	M0	72 (94.7%)
	M1	1 (1.3%)
Tumor stage (TNM)	III	19 (25%)
	IVA	49 (64.5%)
	IVB	7 (9.2%)
	IVC	1 (1.3%)
Performance status (PS)	≤1	70 (92.1%)
	>1	6 (7.9%)
Previous surgery	Yes	40 (52.6%)
	No	36 (47.4%)
Type of treatment	Surgery + RT	29 (38.2%)
	Surgery + CRT	22 (28.9%)
	RTH alone	8 (10.5%)
	Induction CTH + RT	7 (9.2%)
	Concurrent CRT	10 (13.2%)
Alcohol consumption	Yes	34 (44.7%)
	No	42 (55.3%)
Smoking status	Smoker	55 (72.4%)
	Non-smoker	21 (27.6%)
	Current smoker	49 (89.1%)
	Former smoker	6 (10.9%)
Relative expression of miRNA-548L	median (range)	4.94 (0.04–12.80)

Abbreviations: CTH—chemotherapy; CRT—chemoradiotherapy; M—metastatic spread; N—lymph node involvement; RT—radiotherapy; T—tumor site and size; TNM—tumor, node, and metastasis staging.

**Table 2:** Comparisons of the relative expression of miRNA-548L according to OM grade in subsequent weeks of RT.

RT Week	OM Grade	Relative Expression of miRNA-548L	
		Median (IQR)	*p*
I	0 (*n* = 4; 5.3%)	5.07 (2.99–7.60)	0.217
	1 (*n* = 72; 94.7%)	2.92 (1.59–5.34)	
II	1 (*n* = 50; 65.8%)	6.65 (3.81–7.71)	0.202
	2 (*n* = 26; 34.2%)	4.19 (2.76–6.21)	
III	1 (*n* = 30; 39.5%)	5.58 (3.14–7.71)	*0.038*
	2 (*n* = 46; 60.5%)	4.21 (2.94–5.91)	
IV	1 and 2 (*n* = 47; 61.8%)	7.13 (5.53–9.00)	*<0.001*
	3 (*n* = 29; 38.2%)	3.89 (2.31–5.41)	
V	1 and 2 (*n* = 61; 80.3%)	6.65 (5.09–8.62)	*0.039*
	3 (*n* = 15; 19.7%)	3.59 (2.85–6.98)	
VI	1 and 2 (*n* = 58; 76.3%)	7.46 (5.53–9.28)	*<0.001*
	3 (*n* = 18; 23.7%)	3.43 (2.76–6.65)	
VII	1 and 2 (*n* = 49; 64.5%)	7.45 (5.52–9.07)	*<0.001*
	3 (*n* = 27; 35.5%)	3.14 (2.49–6.09)	

Results in italics are statistically significant. Abbreviations: IQR—interquartile range; OM—oral mucositis; RT—radiotherapy.

**Table 3:** Diagnostic accuracy of pretreatment miRNA-548L expression for predicting severe OM during subsequent RT cycles.

RT Week	OM Grade	AUC (95%CI)	Sensitivity (%)	Specificity (%)	Cut-Off Value	*p*
I	0	0.59 (0.47–0.70)	47.8	76.7	<5.91	0.204
	1					
II	0	0.65 (0.53–0.75)	57.7	76	<6.21	0.128
	1 and 2					
III	1	0.67 (0.55–0.78)	80	55.7	<4.92	*0.019*
	2					
IV	1 and 2	0.81 (0.71–0.89)	79.3	74.5	<5.41	*<0.001*
	3					
V	1 and 2	0.77 (0.66–0.85)	94.4	62.1	<4.92	*<0.001*
	3					
VI	1 and 2	0.82 (0.63–0.83)	72.2	100	<1.57	*<0.001*
	3					
VII	1 and 2	0.86 (0.76–0.93)	92.6	73.4	<2.97	*<0.001*
	3					

Results in italics are statistically significant. Abbreviations: AUC—area under the curve; CI—confidence interval; OM—oral mucositis; RT—radiotherapy.

## Data Availability

The original contributions presented in this study are included in this article. Further inquiries can be directed to the corresponding author.

## Supplementary Materials

The following supporting information can be downloaded at: https://www.mdpi.com/article/10.3390/genes17050578/s1, Table S1. Comparisons of the relative expression of miRNA-548L depending on patients’ clinical-demographic factors; Table S2. Influence of demographic, clinical and molecular factors on the risk of more severe OM after subsequent cycles (1–4) of RT; Table S3. Influence of demographic, clinical and molecular factors on the risk of more severe OM after subsequent cycles (5–7) of RT; Table S4. Influence of demographic, clinical, nutritional and epigenetic variables on overall survival; Table S5. Identified pathways and target genes of miRNA-548L involved in three critical processes underlying the etiology of oral mucositis (Based on Reactome and Panther analysis); Table S6. Significantly enriched Gene Ontology (GO) terms associated with genes targeted by miRNA-548L and linked to oral mucositis.
