# Supplementary material for: Circulating Plasma miRNA-548L as Novel Predictive Biomarker of Radiotherapy-Induced Severe Oral Mucositis in Patients with Laryngeal Cancer
Source: Genes (Basel). 2026 May 18;17(5):578. doi: 10.3390/genes17050578 (PMC13205370; doi:10.3390/genes17050578)
Supplement: Supplementary file 1 [file genes-17-00578-s001.zip › genes-4302771-supplementary.pdf]

Table S1. Comparisons of the relative expression of miRNA-548L depending on patients' clinical-demographic factors

Factor		miRNA-548L	
		Median (IQR)	*p*
**Gender**	Male	2.81 (1.72-6.65)	0.281
	Female	5.31 (3.39-7.59)	
**Age**	>63	4.95 (2.95-7.72)	0.884
	≤63	5.21 (2.98-7.25)	
**T category**	T4	4.21 (2.82-7.05)	0.235
	T1-3	5.73 (3.39-6.79)	
**N category**	N1-3	4.23 (2.82-7.46)	0.457
	N0	5.07 (3.50-7.60)	
**Tumor stage (TNM)**	IVA- C	2.96 (238-4.44)	***<0.001***
	III	5.90 (3.51-8.32)	
**Performance status (PS)**	≤1	8.91 (4.86-9.44)	***0.024***
	>1	4.93 (2.94-7.45)	
**Previous surgery**	Yes	4.89 (3.04-7.46)	0.794
	No	5.07 (2.95-8.11)	
**Alcohol consumption**	Yes	4.77 (3.14-7.48)	0.966
	No	5.36 (2.94-7.44)	
**Smoking status**	Smoker	4.44 (3.10-6.69)	0.340
	Non-smoker	5.53 (2.95-8.15)	
**Surgery + RT**	Yes	5.20 (2.95-7.49)	0.546
	No	5.25 (3.48-7.71)	
**Surgery + CRT**	Yes	4.90 (3.14-7.48)	0.903
	No	5.47 (2.99-7.71)	
**RT alone**	Yes	5.20 (3.306-8.41)	0.646
	No	5.53 (3.32-6.65)	
**Induction CTH+RT**	Yes	5.20 (3.06-7.71)	0.987
	No	5.53 (3.22-7.27)	
**Concurrent CRT**	Yes	5.20 (2.95-7.32)	0.835
	No	4.86 (3.08-7.54)	

Abbreviations: CTH – chemotherapy; CRT—chemoradiotherapy; IQR – interquartile range; N – lymph nodeinvolvement; RT—radiotherapy; T—tumor site and size; TNM – tumor, node, and metastasis staging

Table S2. Influence of demographic, clinical and molecular factors on the risk of more severe OM after subsequent cycles (1–4) of RT

Factor	Grade 1 OM after 1st cycle of RT		Grade 2OM after 2nd cycle of RT		Grade 2 OM after 3rd cycle of RT		Grade 3 OM after 4th cycle of RT	
	Univariable	Multivariable	Univariable	Multivariable	Univariable	Multivariable	Univariable	Multivariable
	OR (95%CI) *p*	OR (95%CI) *p*	OR (95%CI) *p*	OR (95%CI) *p*	OR (95%CI) *p*	OR (95%CI) *p*	OR (95%CI) *p*	OR (95%CI) *p*
**Gender** (male)	1.85 (0.17-19.43) 0.608	1.16 (0.07-17.80) 0.915	1.05 (0.28-3.97) 0.944	0.75 (0.18-3.08) 0.693	1.66 (0.48-5.76) 0.419	1.63 (0.40-6.69) 0.494	2.19 (0.54-8.86) 0.271	1.71 (0.25-11.78) 0.589
**Age**(>63)	1.06 (0.14-7.20) 0.956	3.91 (0.21-7.75) 0.356	1.17 (0.45-3.02) 0.750	1.19 (0.44-3.23) 0.736	0.70 (0.28-1.77) 0.451	0.87 (0.32-2.40) 0.783	0.74 (0.29-1.84) 0.513	0.51 (0.14-1.89) 0.311
**T category** (T4)	3.54 (0.35-35.72) 0.282	2.15 (0.05-11.3) 0.705	2.22 (0.83-5.92) 0.111	1.73 (0.61-4.87) 0.299	2.33 (0.91-5.98) ***0.037***	1.85 (0.69-4.98) 0.217	3.00 (1.13-7.96) ***0.027***	1.60 (0.44-5.83) 0.476
**N category** (N1-3)	0.71 (0.07-7.21) 0.772	0.43 (0.04-5.09) 0.525	1.87 (0.64-5.52) 0.253	2.13 (0.68-6.69) 0.191	1.15 (0.43-3.06) 0.790	1.03 (0.35-3.08) 0.954	0.87 (0.33-2.34) 0.791	0.74 (0.18-3.01) 0.671
**Tumor stage (TNM)** (IVA-C)	3.23 (0.43-24.73) 0.257	0.42 (0.02-9.13) 0.582	6.18 (1.03-29.32) ***0.021***	3.91 (0.76-20.13) 0.102	2.75 (0.94-7.97) 0.062	1.02 (0.28-3.72) 0.970	7.37 (2.17-139.05) ***0.007***	3.15 (0.32-31.62) 0.329

**Performance status (PS)**(≤1)	0.89 (0.04-18.22) 0.933	1.89 (0.19-10.45) 0.998	0.36 (0.04-3.26) 0.363	0.26 (0.03-2.53) 0.247	0.29 (0.05-1.73) 0.175	0.28 (0.03-1.57) 0.140	3.38 (0.58-19.78) 0.175	18.87 (1.14-311.11) ***0.039***
**Previous surgery** (yes)	0.35 (0.03-3.55) 0.376	0.47 (0.03-5.69) 0.549	0.53 (0.20-1.39) 0.196	0.44 (0.15-1.22) 0.113	0.76 (0.30-1.93) 0.569	1.05 (0.36-3.09) 0.927	0.54 (0.21-1.36) 0.191	0.43 (0.11-1.72) 0.231
**Alcohol consumption**(yes)	2.54 (0.25-25.57) 0.429	1.58 (0.11-20.97) 0.726	1.75 (0.67-4.55) 0.251	1.78 (0.65-4.89) 0.261	1.37 (0.54-3.49) 0.502	1.44 (0.50-4.14) 0.494	2.23 (0.87-5.70) ***0.033***	2.24 (0.62-8.11) 0.220
**Smoking status**(smoker)	0.86 (0.08-8.83) 0.903	1.60 (0.09-29.76) 0.753	1.43 (0.48-4.27) 0.523	1.14 (0.35-3.62) 0.833	1.21 (0.44-3.37) 0.709	1.17 (0.38-3.55) 0.791	1.93 (0.65-5.74) 0.233	1.31 (0.31-5.59) 0.717
**Surgery + RT** (yes)	1.25 (0.17-9.37) 0.828	0.88 (0.09-7.81) 0.908	2.43 (0.90-6.63) ***0.031***	2.84 (0.97-8.28) 0.055	2.23 (0.87-5.70) ***0.013***	2.59 (0.94-7.13) 0.064	1.73 (0.67-4.43) 0.255	3.32 (0.80-13.81) 0.098
**Surgery + CRT** (yes)	0.49 (0.05-4.99) 0.550	0.52 (0.03-7.89) 0.635	0.65 (0.25-1.72) 0.391	1.81 (0.64-5.01) 0.261	0.40 (0.15-1.07) 0.068	1.73 (0.48-6.20) 0.396	0.48 (0.19-1.24) 0.132	4.56 (1.01-20.54) ***0.048***
**RT alone** (yes)	1.17 (0.11-11.99) 0.896	0.26 (0.01-6.18) 0.398	1.93 (0.55-6.67) 0.297	0.52 (0.14-1.92) 0.326	2.03 (0.68-6.06) 0.201	0.42 (0.12-1.38) 0.153	1.25 (0.41-3.86) 0.689	0.46 (0.10-2.09) 0.317
**Induction CTH+RT** (yes)	0.54 (0.03-10.66) 0.682	0.85 (0.35-1.28) 0.997	0.34 (0.09-1.25) 0.104	0.28 (0.07-1.20) 0.087	1.21 (0.33-4.44) 0.768	1.76 (0.42-7.27) 0.434	0.71 (0.19-2.61) 0.610	2.45 (0.37-16.07) 0.350

**Concurrent CRT** (yes)	0.37 (0.02-7.24) 0.511	0.97 (0.06-11.89) 0.893	0.67 (0.21-2.18) 0.507	0.82 (0.23-2.87) 0.755	2.62 (0.81-8.45) 0.105	6.44 (1.51-27.58) ***0.012***	0.32 (0.10-1.05) 0.060	0.68 (0.14-3.23) 0.630
**Relative expression of miRNA-548L** (≤4.94)	3.35 (1.33-13.78) 0.304	1.59 (1.09-8.39) 0.753	2.68 (1.04-6.94) ***0.041***	2.33 (1.85-6.38) 0.099	4.07 (1.44-11.46) ***0.007***	4.01 (2.39-9.49) ***0.009***	11.43 (3.63-26.00) ***<0.001***	9.44 (2.44-26.54) ***0.001***

Abbreviations: CI – confidence interval; CRT—chemoradiotherapy; CTH – chemotherapy; N – lymph node involvement; OM –oral mucositis; OR – odds ratio; RT – radiotherapy; T—tumor site and size; TNM – tumor, node, and metastasis staging

Table S3. Influence of demographic, clinical and molecular factors on the risk of more severe OM after subsequent cycles (5–7) of RT

Factor	Grade 3 OM after 5th cycle of RT		Grades3 OM after 6th cycle of RT		Grade 3 OM after 7th cycle of RT	
	**Univariable**	**Multivariable**	**Univariable**	**Multivariable**	**Univariable**	**Multivariable**
	OR (95%CI) *p*	OR (95%CI) *p*	OR (95%CI) *p*	OR (95%CI) *p*	OR (95%CI) *p*	OR (95%CI) *p*
**Gender** (male)	3.08 (0.36-25.94) 0.300	3.95 (0.36-43.13) 0.259	0.56 (0.14-2.13) 0.395	0.11 (0.01-1.36) 0.084	1.12 (0.30-4.14) 0.862	0.59 (0.06-5.53) 0.645
**Age**(>63)	0.57 (0.18-1.79) 0.331	0.71 (0.17-2.92) 0.635	1.25 (0.43-3.62) 0.680	1.12 (0.27-4.59) 0.874	0.82 (0.33-2.10) 0.681	0.77 (0.15-3.84) 0.750
**T category** (T4)	2.06 (0.63-6.76) 0.229	2.39 (0.54-10.59) 0.248	2.14 (0.71-6.48) 0.177	0.97 (0.22-4.27) 0.971	2.45 (0.92-6.53) ***0.072***	1.17 (0.24-5.63) 0.848
**N category** (N1-3)	2.10 (0.53-8.28) 0.288	1.64 (0.36-7.45) 0.521	0.65 (0.22-1.96) 0.446	0.51 (0.10-2.58) 0.415	0.88 (0.32-2.40) 0.807	0.76 (0.12-4.89) 0.772
**Tumor stage (TNM)** (IVA-C)	14.22 (0.80-250.05) 0.069	21.71 (0.72-197.11) 0.993	7.65 (0.94-61.98) ***0.046***	0.32 (0.02-5.34) 0.426	5.09 (1.88-12.85) ***0.010***	2.52 (0.14-44.13) 0.527

**Performance status (PS)**(≤1)	0.80<br>(0.08-7.41)<br>0.844	0.46<br>(0.03-7.09)<br>0.582	1.68<br>(0.28-10.07)<br>0.556	2.63<br>(0.22-31.31)<br>0.445	0.90<br>(0.15-5.26)<br>0.906	1.71<br>(0.10-29.71)<br>0.711
**Previous surgery**<br>(yes)	0.53<br>(0.17-1.67)<br>0.278	0.91<br>(0.23-3.65)<br>0.893	0.65<br>(0.22-1.88)<br>0.427	0.57<br>(0.13-2.55)<br>0.459	0.95<br>(0.37-2.44)<br>0.919	3.23<br>(0.51-20.35)<br>0.211
**Alcohol consumption**(yes)	1.54<br>(0.49-4.78)<br>0.456	3.32<br>(0.73-15.09)<br>0.121	0.98<br>(0.34-2.85)<br>0.977	0.76<br>(0.19-3.09)<br>0.702	1.24<br>(0.48-3.18)<br>0.657	0.92<br>(0.20-4.26)<br>0.925
**Smoking status**(smoker)	1.67<br>(0.42-6.65)<br>0.464	2.71<br>(0.49-15.06)<br>0.253	2.25<br>(0.56-8.75)<br>0.242	1.29<br>(0.21-7.89)<br>0.785	1.54<br>(0.52-4.60)<br>0.435	1.25<br>(0.18-8.57)<br>0.820
**Surgery + RT**<br>(yes)	2.66<br>(0.76-9.28)<br>0.124	1.32<br>(0.25-6.87)<br>0.740	1.37<br>(0.46-4.03)<br>0.568	1.69<br>(0.39-7.32)<br>0.480	2.08<br>(0.78-5.53)<br>0.140	3.97<br>(0.66-23.67)<br>0.130
**Surgery + CRT**<br>(yes)	0.24<br>(0.07-0.81)<br>***0.021***	4.47<br>(1.12-17.91)<br>***0.034***	1.03<br>(0.34-3.05)<br>0.953	0.96<br>(0.22-4.17)<br>0.956	0.57<br>(0.22-1.48)<br>0.252	2.10<br>(0.40-11.00)<br>0.378
**RT alone**<br>(yes)	1.19<br>(0.29-4.83)<br>0.806	0.46<br>(0.08-2.46)<br>0.364	0.68<br>(0.20-2.28)<br>0.529	2.38<br>(0.44-12.81)<br>0.312	1.01<br>(0.33-3.13)<br>0.981	0.96<br>(0.17-5.28)<br>0.964
**Induction CTH+RT**<br>(yes)	0.70<br>(0.03-14.53)<br>0.818	0.25<br>(0.05-1.37)<br>0.109	0.49<br>(0.12-1.95)<br>0.315	0.65<br>(0.09-4.84)<br>0.675	0.66<br>(0.18-2.43)<br>0.536	3.01<br>(0.34-28.85)<br>0.324

**Concurrent CRT** (yes)	0.54 (0.14-2.07) 0.367	0.97 (0.21-4.57) 0.973	0.56 (0.16-1.94) 0.360	1.11 (0.21-6.01) 0.900	0.60 (0.19-1.92) 0.391	6.11 (0.52-71.19) 0.148
**Relative expression of miRNA-548L** (≤4.94)	5.03 (1.29-13.67) ***0.020***	5.02 (1.06-23.75) ***0.042***	7.82 (3.46-22.38) ***0.001***	11.57 (2.44-19.72) ***0.005***	8.28 (2.66-25.78) ***<0.001***	10.35 (1.76-26.74) ***0.009***

Abbreviations: CI – confidence interval; CRT—chemoradiotherapy; CTH – chemotherapy; N – lymph node involvement; OM –oral mucositis; OR – odds ratio; RT – radiotherapy; T—tumor site and size; TNM – tumor, node, and metastasis staging

Table S4. Influence of demographic, clinical, nutritional and epigenetic variables on overall survival

Variable		Overall survival				
		Univariable			Multivariable	
		mOS (months)	HR (95% CI)	*p*	HR (95% CI)	*p*
**Gender**	Men	34	1.19 (0.49-2.88)	0.711	0.85 (0.33-2.21)	0.741
	Women	36	0.84 (0.35-2.04)			
**Age** (years)	<63	39	0.86 (0.45-1.66)	0.662	1.04 (0.54-2.01)	0.895
	≥63	34	1.15 (0.59-2.01)			
**T category**	T4	19	3.1 (1.61-5.99)	***<0.001***	2.01 (0.88-4.59)	***0.045***
	T1-3	38	0.32 (0.17-0.62)			
**N category**	N1-3	32	1.27 (0.63-2.52)	0.513	0.69 (0.33-1.46)	0.341
	N0	34	0.79 (0.39-1.58)			
**Tumor stage (TNM)**	IVA- C	19	3.75 (2.43-9.28)	***<0.001***	2.71 (1.99-22.59)	***0.002***
	III	34	0.21 (0.11-0.41)			
**Performance status (PS)**	≤1	19	1.29 (0.41-4.11)	0.611	1.05 (0.37-2.99)	0.931
	>1	34	0.77 (0.24-2.44)			
**Previous surgery**	Yes	29	1.35 (0.71-2.56)	0.355	1.64 (0.84-3.21)	0.147
	No	34	0.74 (0.39-1.42)			
**Alcohol consumption**	Yes	25	1.48 (0.76-2.86)	0.219	1.32 (0.69-2.54)	0.395
	No	36	0.67 (0.35-1.31)			
**Smoking status**	Smoker	25	1.78 (0.88-3.62)	0.150	1.91 (0.84-4.36)	0.124
	Non-smoker	32	0.56 (0.28-1.14)			
**Surgery + RT**	Yes	38	1.52 (0.27-0.99)	0.137	0.45 (0.23-0.92)	***0.031***
	No	19	1.91 (1.01-3.69)			
**Surgery + CRT**	Yes	25	1.44 (0.74-2.85)	0.244	1.36 (0.70-2.66)	0.364
	No	36	0.69 (0.35-1.36)			
**RT alone**	Yes	36	0.56 (0.26-1.18)	0.180	0.57 (0.24-1.37)	0.215
	No	34	1.78 (0.85-3.72)			
**Induction CTH+RT**	Yes	19	1.38 (0.52-3.70)	0.455	0.78 (0.32-1.89)	0.592
	No	34	0.72 (0.27-1.94)			
**Concurrent CRT**	Yes	23	0.79 (0.34-1.79)	0.584	0.39 (0.61-3.58)	0.389
	No	34	1.26 (0.56-2.87)			
**Relative expression of miRNA-548L**	Low (<4.94)	18	3.12 (1.09-4.12)	***0.010***	1.64 (0.83-3.25)	***0.026***
	High (≥4.94)	36	0.47 (0.25-0.92)			

Abbreviations:CI – confidence interval; CTH – chemotherapy; CRT—chemoradiotherapy; HR – hazard ratio; N – lymph node involvement; OS – overall survival; RT—radiotherapy; T—tumor site and size; TNM – tumor, node, and metastasis staging

Table S5. Identified pathways and target genes of miRNA-548L involved in three critical processes underlying the etiology of oral mucositis (Based on Reactome and Panther analysis)

Description	Pathway ID	Gene	adjusted p-value	Category	Link to oral mucositis
**Histamine H1 receptor mediated signaling pathway**	P04385	**GNG12**	0.0299	**Inflammation**	H1 activation leads to vasodilation, increased vascular permeability → edema and erythema of oral mucosa
**Histamine H2 receptor mediated signaling pathway**	P04386				H2 activation modulates immune response in mucosal tissues
**Beta1 adrenergic receptor signaling pathway**	P04377				Adrenergic receptors regulate stress responses, epithelial cell survival, and inflammation
**Beta2 adrenergic receptor signaling pathway**	P04378				
**Beta3 adrenergic receptor signaling pathway**	P04379				
**Cortocotropin releasing factor receptor signaling pathway**	P04380				Chronic stress via CRF can increase pro-inflammatory cytokines, exacerbating mucosal damage
**5HT1 type receptor mediated signaling pathway**	P04373			**Pain signaling**	Serotonin can modulate pain (nociceptor sensitization) → oral pain in mucositis
**5HT2 type receptor mediated signaling pathway**	P04374		0.0342		

**5HT4 type receptor mediated signaling pathway**	P04376		0.0299		Dysregulated 5HT signaling may exacerbate mucosal inflammation or reduce epithelial repair
**Opioid proopiomelanocortin pathway**	P05917				Impaired opioid signaling → increased pain perception.
**Opioid proenkephalin pathway**	P05916				Opioids can modulate local immune responses, so dysregulation may worsen mucosal injury and delay healing
**Enkephalin release**	P05913				
**Oxytocin receptor mediated signaling pathway**	P04391		0.0330	**Epithelial repair**	Reduced oxytocin signaling may impair mucosal healing and increase inflammation
**Thyrotropin-releasing hormone receptor signaling pathway**	P04394				Dysregulated TRH signaling may alter epithelial turnover in oral mucosa → delayed recovery
**Beta adrenergic receptor signaling pathway**	P04377, P04378, P04379		0.0299		Beta2 stimulation can modulate cytokine release and tissue repair<br><br>Beta3 is involved in vascular regulation and may influence mucosal blood flow
**SUMOylation of RNA binding proteins**	R-HSA-4570464	**NUP160**<br><br>**BMI1**	0.0421		Affects expression of genes critical for stress response, inflammation, and epithelial proliferation<br><br>Dysregulation can amplify tissue damage or slow repair
**SUMOylation of DNA damage response and repair proteins**	R-HSA-3108214				Oral mucositis is often triggered by DNA damage in basal epithelial cells from chemo/radiotherapy

					Impaired SUMOylation → reduced repair → more cell death, ulceration, delayed healing

Table S6. Significantly enriched Gene Ontology (GO) terms associated with genes targeted by miRNA-548L and linked to oral mucositis

GO category	GO term	Term ID	Gene	adjusted p-value	Link to oral mucositis
**Biological process**	Regulation of wound healing	GO:0061041	**SRSF6**	0.0244	Oral mucositis is fundamentally a disorder of impaired mucosal healing
	Regulation of keratinocyte proliferation	GO:0010837			Keratinocyte proliferation in the basal layer is essential for regeneration after chemo/radiation injury
	Epithelial cell apoptotic process	GO:1904019			Chemo- and radiotherapy induce epithelial apoptosis, initiating mucositis
	Negative regulation of apoptotic signaling pathway	GO:2001234	**BMI1**	0.0438	Excessive epithelial cell death → more severe mucositis
	Negative regulation of keratinocyte differentiation	GO:0045617	**SRSF6**	0.0244	Dysregulation can weaken mucosal barrier → increased inflammation and ulcer risk
**Cellular process**	No significant identified	-	**-**	-	-
**Molecular function**	Ubiquitin-protein transferase activator activity	GO:0097027	**BMI1**	0.0475	Ubiquitination regulates: NF-κB signaling, p53-mediated apoptosis, DNA damage response
